# Interventions to increase migrants’ care-seeking behaviour for stigmatised conditions: a scoping review

**DOI:** 10.1007/s00127-021-02065-1

**Published:** 2021-03-29

**Authors:** Vanessa Place, Benjamin Nabb, Karima Viksten Assel, Sofie Bäärnhielm, Christina Dalman, Anna-Clara Hollander

**Affiliations:** 1 Karolinska Institutet, Stockholm, Sweden; 2 Transkulturellt Centrum, Stockholm, Sweden; 3 Centre for Epidemiology and Community Medicine, Stockholm, Sweden; 4 Research Group Epidemiology of Psychiatric Conditions, Substance Use and Social Environment (EPICSS), Department of Public Health Sciences, Karolinska Institutet, Solnavägen 1E, SE-171 77 Stockholm, Sweden

**Keywords:** Migrants, High-income countries, Stigma, Scoping review, Interventions to increase care-seeking behaviour, Mental health, TB, HIV, Hepatitis, Care-seeking

## Abstract

**Background:**

Despite availability of effective treatments, migrants in high-income countries seek care for conditions associated with stigma to a lower extent than the rest of the population. We conducted a scoping review to map the literature on interventions to increase migrants’ care-seeking behaviour in high-income countries for stigmatised conditions.

Main body of the abstract: We searched 15 electronic databases and journals, hand-searched references and citations, to identify studies on interventions to increase migrants’ care-seeking in high-income countries for stigmatised conditions. We applied language restrictions for English and Swedish, and searched the full time period up to 5 July 2019. Our primary outcome of interest was care utilisation.

**Results:**

5447 records were identified in the literature searches. We identified 16 eligible studies, all from North America, that reported interventions to increase migrants’ care-seeking behaviour for hepatitis B (*n* = 1) and mental health (*n* = 15). Three approaches were identified: health communication (*n* = 10), support groups (*n* = 2), and primary care-based approaches (*n* = 4). There was a general trend towards community-based interventions tailored to individual migrant groups. Significant gaps were identified in the literature, including studies conducted in Europe and studies including men or children. Furthermore, the choice of study designs introduced significant bias that prevented accurate conclusions on intervention effectiveness.

**Conclusion:**

The available evidence on interventions to increase migrants’ in high-income countries care-seeking behaviour for stigmatised conditions is limited in scope and quality. Future research, using reliable study designs, is needed to fill the remaining gaps and to boost the scope and reliability of the evidence.

**Supplementary Information:**

The online version contains supplementary material available at 10.1007/s00127-021-02065-1.

## Background

Over recent decades, international migration has reached unprecedented levels [1]. The number of international migrants—defined by the International Organisation for Migration as individuals living outside their country of birth—reached an estimated 272 million in 2019 [2]. While globalisation has made migration easier and cheaper, a host of other factors including conflict, poverty, and adverse effects of climate change have contributed to increasing numbers of people forced to leave their homes.

The upward trend in migration has coincided with growing recognition of migration as a social determinant of health [3, 4]. The combination of socioeconomic, environmental, and political factors that migrants are exposed to pre-, during, and post-migration interact to impact their health [5]. Given the heterogeneity of circumstances under which migration occurs [2], the range of health risks and protective factors that migrants are exposed to is broad [5].

Although generalisations about migrants should be made with caution, migrants face cultural, legal, and socioeconomic barriers to accessing healthcare in their host countries [6, 7]. Commonly reported barriers include communication difficulties, cultural differences leading to dissatisfaction with services, discrimination, and issues obtaining care without permanent status [8].

Care-seeking behaviour has been defined as “a problem-focused, planned behaviour, involving interpersonal interaction with a selected healthcare professional” [9]. This definition does not include care-seeking from informal sources, such as traditional healers or social networks, which are also important forms of care-seeking within many communities [10]. Several studies have demonstrated that migrant groups in high-income countries (HICs) exhibit generally lower care-seeking behaviour from health professionals than the wider population [11–14]. The reasons behind this are not well understood, but are likely linked to the barriers to care [6, 7] and even care-seeking from informal sources [10].

Various health conditions, such as sexually transmitted infections (STIs) including HIV [15], and mental health problems such as depression [16], are associated with stigma. Stigma is defined by the Cambridge Dictionary as “a strong feeling of disapproval that most people in society have about something”, and these stigmatised conditions are often associated with barriers to care in general populations [16, 17]. As migrant populations are often themselves stigmatised within society [18], they likely face further barriers to seeking care for stigmatised conditions than the rest of the population. One qualitative study with sub-Saharan African immigrants in Australia, for example, identified fear of rejection within the community as a barrier to seeking mental health care [19]. Indeed, care-seeking for stigmatised conditions is low amongst migrant groups [7].

The availability of effective treatments for many stigmatised conditions has increased [15, 20], particularly in high-income countries. However, barriers to care and subsequent low care-seeking prevent migrant populations from accessing these vital services. Delaying or not seeking care has been associated with poorer health outcomes [21] and can widen health disparities between vulnerable groups and the wider population [7]. Improving migrants’ care-seeking behaviour for stigmatised conditions in high-income countries could, therefore, has a significant impact on migrant health.

Interventions implemented to improve migrant health and care-seeking have been previously documented [22]. Prior to this review, however, the scope of the evidence on interventions implemented to increase migrants’ care-seeking for stigmatised conditions in high-income countries was unknown. A scoping review approach was therefore deemed appropriate for this study [23]. The objectives were to assess the scope of the literature and to gather knowledge on intervention features and outcomes. The overarching purpose was to inform the design of an intervention to increase migrants’ care-seeking behaviour for children and young peoples’ mental health services in Stockholm, Sweden.

### Scoping review question

Which interventions to increase migrants’ care-seeking behaviour for stigmatised conditions have been implemented in high-income countries?

## Methodology

### Protocol and registration

Two reviewers (VP & BN) developed the review protocol using PRISMA-ScR guidance on conducting scoping reviews [23], based upon Arksey and O’Malley’s methodological framework and later guidance [24, 25]. The protocol was modified following literature searches and feedback from the research team. The final version, and the PRISMA-ScR checklist, are included as appendices (Appendix 1 and 2).

### Eligibility criteria

Studies that met the following criteria were considered: study design included an intervention that was implemented in a high-income country (using the World Bank’s Classification of Countries by Income [26], from the year that each study was conducted); the intervention was designed to increase initial care-seeking behaviour from a healthcare professional for a stigmatised condition; the main study population was international migrants, and/or their children. Studies that compared outcomes between migrant groups and the wider population were included. Studies that targeted a vulnerable subgroup (such as homeless migrants) were excluded, as preliminary literature searches indicated that these studies focussed on lowering barriers associated with subgroup, not migrant, status. Studies conducted in low- or middle-income countries were beyond the scope of this review and were excluded.

The focus of this scoping review was the initial care-seeking act (the first time an individual seeks professional help for a health problem), thus interventions that sought to retain patients in care were not included. Interventions to improve adherence to a treatment regimen, or to promote screening or vaccination, were excluded. Interventions that sought to improve attitudes towards care-seeking, or willingness to seek care, were included.

Moreover, the purpose of this study was to inform the design of an intervention to increase care-seeking for mental health problems amongst migrant populations. Prior to conducting this scoping review, the number of studies reporting interventions to improve care-seeking for mental health problems was anticipated to be low, thus interventions to improve care-seeking for any stigmatised condition were included. All years, study designs, and publication types were considered for inclusion, including peer-reviewed and grey literature.

### Information sources and search strategy

The authors collaborated with information experts at Karolinska Institutet University Library to develop a comprehensive search strategy that was continually revised. Searches of 13 electronic databases and websites, covering peer-reviewed and grey literature (PubMed; Web of Science; PsycINFO; Global Health; Google Scholar; Mednar; ProQuest; DART-Europe; OAIster; Bielefeld Academic Search Engine (BASE); National Center for Biotechnology Information (NCBI) Bookshelf; the World Health Organisation; Norwegian Institute of Public Health), as well as hand-searches of key journals within the field (Ethnicity & Health; BMC Public Health, selected by VP and ACH), were conducted from 10/06/2019 until 05/07/2019. The search strategy for PubMed is presented Supplementary Fig. 1; other search strategies are available upon request. The search was limited to studies published in English or Swedish. Manual searches of citations on Google Scholar (citation tracking) and references lists (reference scans) were conducted to identify additional studies for review.

### Selection of sources of evidence

Study selection was an iterative process, and the search strategy was modified following new findings in the literature. Search results were imported into Mendeley reference management software and screened against the eligibility criteria. Titles and abstracts were screened independently by VP and BN, who met to decide which studies were eligible for full-text screening. Full-text screens of these studies were conducted independently by VP and BN, who then decided the final list of studies for inclusion. Disagreements were resolved by consultation with the research team.

### Data items and the data charting process

The following data were extracted from studies: (i) author(s), publication year, location; (ii) study population (e.g. ethnic group); (iii) study design; (iv) intervention characteristics (e.g. language); (v) stigmatised condition(s); and (vi) reported outcomes related to the scoping review question (e.g. psychiatric service utilisation), using a custom-built data extraction form.

The data extraction form was developed by VP and independently piloted on three included studies by VP and BN. The reviewers then discussed the form with the research team, using feedback to adjust as necessary. As far as possible, reviewers sought to extract data as reported in the studies. Methodological details that were not explicitly stated, such as sampling method, were inferred from the manuscript.

### Synthesis of results

Descriptive data from included studies were summarised in text and table format (Table 1). To synthesise results on the type of interventions reported in included studies, qualitative synthesis using qualitative content analysis [27] was employed. Reported interventions were organised thematically, into themes corresponding to the approach taken to increase care-seeking behaviour by VP, BN and ACH. This was an iterative process, using the intervention type as the primary unit of analysis and involving discussion with the research group to address potential biases and preconceptions of the reviewers.

**Table 1:** Key characteristics of included studies (*n* = 16)

Intervention approach	Author(s), publication year, location	Stigmatised condition	Study population	Study design	Intervention description	Results
Health Communication						
*Entertainment Education (EE)*	Hernandez & Organista, 2013 USA^1^	Mental health (depression)	Latina migrants; 79·0% born in Mexico	Two-group pre/post-test randomised controlled trial	*Secret Feelings*: a fotonovela written in Spanish at 4th grade reading level that presents the story of a depressed middle-aged Latina mother. The control group were exposed to a discussion of family communication and inter-generational relationships. 2–12 participants per group	Marginally statistically significant difference (p = 0·012) in the mean increase in intent to seek treatment for depression between the intervention and control group, pre- to post-intervention
	Unger et al., 2013 USA^2^	Mental health (depression)	Hispanics (84·0% migrants)	Longitudinal randomised controlled trial	The intervention group were exposed to the fotonovela *Secret Feelings* while the control group were exposed to a low-literacy pamphlet about depression. The intervention was implemented at a community adult school	No statistically significant difference in willingness to seek help for depression pre- to post-intervention or at one-month follow up, in the intervention or control group. The difference between the two groups was also not significant
	López et al., 2009 USA^3^	Mental health (psychosis)	Spanish-speaking Latinxs (86·0% migrants)	One-group pre/post-test	*LA CLAve*: a 35-min psychoeducational program including PowerPoint slides, audio, videos, and art (in Spanish), intended to increase psychosis literacy. Participants were community residents or caregivers of people with schizophrenia and received the intervention in groups of 9–30	Amongst community residents there was a statistically significant (*p* = 0·008) increase in the likelihood to recommend professional help; and a statistically significant (*p* < 0·001) decrease in the likelihood to recommend personal solutions, for the symptoms of psychosis, pre- to post-intervention. No significant difference in likelihood to recommend personal (* p* = 1·00) or professional help (* p* = 0·79) amongst caregivers, pre- to post-intervention
	Casas et al., 2014 USA^4^	Mental health (psychosis)	Spanish-speakers(86·0% migrants)	One-group pre/post-test	*LA CLAve*: a 35-min psychoeducational program to increase psychosis literacy, administered in DVD format by a community mental health educator	Statistically significant (* p* < 0·001) increase in participants’ recommendations of professional help-seeking, and a statistically significant (* p* < 0·001) decrease in recommendations for use of social resources for the symptoms of psychosis, pre- to post-intervention
	Heilemann et al., 2017 USA^5^	Mental health (depression and anxiety)	Latinas	One-group pre/post-test	*Catalina: Confronting my Emotions*: participants used their personal devices to engage in a web-based transmedia intervention (in English) that included: i) story-based videos; ii) a data-informed psychotherapeutic video; iii) an interactive video sequence; and iv) a blog with links to mental health resources	39·0% and 28·5% of participants reported using the online resources within one and six weeks of intervention exposure, respectively. Participants' level of perceived confidence in their ability to seek help was significantly associated with intention/action to seek help at one (* p* = 0·005) and six weeks (* p* = 0·04) post-exposure. Perceived importance of seeking help was significantly associated with intention/action to seek help at one (* p* = 0·009) and six weeks (* p* = 0·003) post-exposure
*Community outreach*	Chao et al., 2007 USA^6^	HBV and liver cancer	Asian-Americans(94·0% migrants)	One-group practice-based study	One-day clinic with free HBV screening and physician-led educational seminars in English and Mandarin. Participants received their test results, alongside a detailed interpretation letter and specific recommendations for follow-up health actions, four weeks later	At one-year follow-up, 67·0% of participants who had screened positive for chronic HBV infection reported that they had followed recommendations to seek liver cancer screening from a physician
	Teng & Friedman, 2009 USA^7^	Mental health	Chinese-Americans	One-group pre/post-test	*Mental Health Awareness Among Older Chinese Adults: The Relationship Between Mind and Body*: a 60-min presentation given in English and translated into Mandarin. It included information on relevant psychiatric disorders, the types of mental health professionals available/services offered	Statistically significant increase (* p* = 0·005) in the inclination to seek help from a mental health professional for psychiatric concerns, pre– to post-intervention
	Tran et al., 2014 USA^8^	Mental health (depression and stress)	Latina migrants; 65·0% Mexican	One-group pre/post-test	ALMA (*Amigas Latinas Motivando el Alma/Latina Friends Motivating the Soul*): Latinas trained as lay health educators (promotoras) who provide women in their social network (compañeras) with support and coping techniques	Statistically significant (* p* < 0·001) increase in positive attitudes towards depression treatment amongst compañeras, pre- to post-intervention
*Non-specific approaches*	Dueweke & Bridges, 2016 USA^9^	Mental health (suicide)	Latinx migrants; 85·0% Mexican	Two-group pre/post-test randomised controlled trial	The intervention group were exposed to a National Institute of Mental Health brochure on suicide, available in English and Spanish. It included prevalence statistics, risk factors, prevention methods, and crisis help lines. The control group were exposed to an information brochure about walking	No significant difference (* p* = 0·17) between the intervention and control group in the change in attitude towards seeking professional psychosocial help pre- to post-intervention
	Piwowarczyk et al., 2013 USA^10^	Mental health (trauma)	Congolese and Somali migrants	One-group pre/post-test	The UJAMBO program: one-session group workshops centred around a DVD that uses African women’s stories to convey information about mammographies, pap smears, and trauma. 4–12 participants per group	Statistically significant increase in participants’ knowledge of available mental health services (* p* = 0·001) and intent to obtain mental health care (* p* < 0·001), pre- to post-intervention
Support groups	Weine et al., 2008 USA^11^	Mental health (PTSD)	Bosnian-Herzegovinian refugees	Longitudinal randomised controlled trial	CAFES (*Coffee and Family Education and Support*): nine multiple-family group sessions over 16 weeks with approximately seven families per group. Primary subjects with PTSD and their family members were invited to attend. The control group received no intervention	On average, individuals in the intervention group attended more mental health visits in the 18 months post-intervention (* n* = 6·04) than the control group (* n* = 1·75). This difference was statistically significant (* p* < 0·049), the effect was constant over all time points (6, 12 and 18 months), and observed in both primary subjects and family members
	Weine et al., 2003 USA^12^	Mental health (trauma)	Kosovar refugees	Two-group quasi-experimental	TAFES (*Tea and Family Education and Support*): a family support and education intervention involving six-session, multi-family groups over eight weeks. Groups were facilitated by trained Kosovar migrants in community settings, with up to six families per group	Statistically significant increase in engagers´ (family members who attended at least one session) use of psychiatric services from baseline to three-month follow-up (* p* < 0·001). There was a small, but non-significant (* p* < 0·166), increase in non-engagers use of psychiatric services over the same period
Primary care	Ahmad et al., 2012 Canada^13^	Psychosocial health	Afghan refugees	Randomised controlled trial	The intervention group were exposed to the CaPRA (*Computer-assisted Psychosocial Risk Assessment*) survey, a self-assessment survey in Dari/Farsi completed in a community health centre waiting room. Patients then received a recommendation sheet summarising their disclosed risks in simple language, while medical providers received a risk-report that summarised patients’ risks with possible referrals. The control group received care as usual	In an exit survey, intention to visit a psychosocial counsellor was higher in the intervention (72·0%) than in the control (46·0%) group, but this difference was not statistically significant (* p* = 0·06)
	Yeung et al., 2004 USA^14^	Mental health	Asian-American migrants	One-group practice-based study	Four-component intervention to integrate psychiatry and primary care. It included: i) training of PCPs on treatment guidelines for common mental disorders; ii) training PCPs and nurses on cultural sensitivity; iii) a primary care nurse who acted as a”bridge” or care manager; and iv) a liaison psychiatrist providing on-site services	Statistically significant increase in the number of patients referred to mental health services (* p* < 0·05) and rates of engagement with these services (* p* < 0·001) during the 12-month duration of the project in comparison to the previous 12 months
	White et al., 2015 USA^15^	Mental health (trauma)	Somali and Ethiopian refugees	One-group quasi-experimental retrospective	Five-component intervention at an urban primary care clinic to increase multi-problem patients’ engagement with treatment, including: i) integrated physical and mental health services; ii) trained interpreters and bicultural health workers; iii) a four-visit protocol to address physical and psychological complaints by primary care providers; iv) co-management of patients receiving physical and mental health services; and v) availability of trauma-informed psychotherapy	The intervention was associated with high levels of adherence to mental health referral (48·0%). Both therapy adherents (patients who engaged in psychotherapy) and non-engagers showed increased primary care utilisation pre– to post-intervention, but the difference between these groups was not statistically significant. No significant difference in both therapy adherents and non-adherents’ usage of emergency and urgent care, pre– to post-intervention
	Ngo et al., 2009 USA^16^	Mental health (depression)	67·0% Latinx, 19·0% black, 14·0% white (55·0% second-generation migrants)	Multi-site randomised controlled trial	Multi-component, multi-site intervention to improve mental health care in primary care. It included: i) expert-led teams that adapted/implemented the intervention at each site; ii) care managers supporting primary care providers; iii) training for care managers in manualized CBT for depression; and iv) patient and provider choice of treatment modalities. The control group had access to care as usual	Statistically significant difference between intervention and control group in: use of psychotherapy/counselling amongst blacks (* p* = 0·036); use of speciality mental health care amongst Latinxs (* p* = 0·046) and blacks (* p* = 0·014); and the number of psychotherapy or counselling visits amongst blacks (* p* = 0·025), from baseline to six-month follow-up. No significant difference between intervention and control group for all ethnicities for the use of mental health care by a primary care clinician (p > 0·05), or for use of any medication (p > 0·05), from baseline to six-month follow-up

*CBT* Cognitive Behavioural Therapy, *HBV* Hepatitis B Virus, *PCP* Primary Care Physician, *PTSD* Post-traumatic Stress Disorder

Several subthemes, representing approaches taken to increase care-seeking, emerged from the overarching themes. Interventions within each theme were further categorised, where appropriate, into these subthemes. Themes and subthemes were discussed between the researchers until a consensus was reached.

### Critical appraisal

Consistent with PRISMA-ScR guidance on conducting scoping reviews [23], the methodological quality and risk of bias of the included studies were not assessed.

## Results

### Selection of sources of evidence

**Fig. 1:**
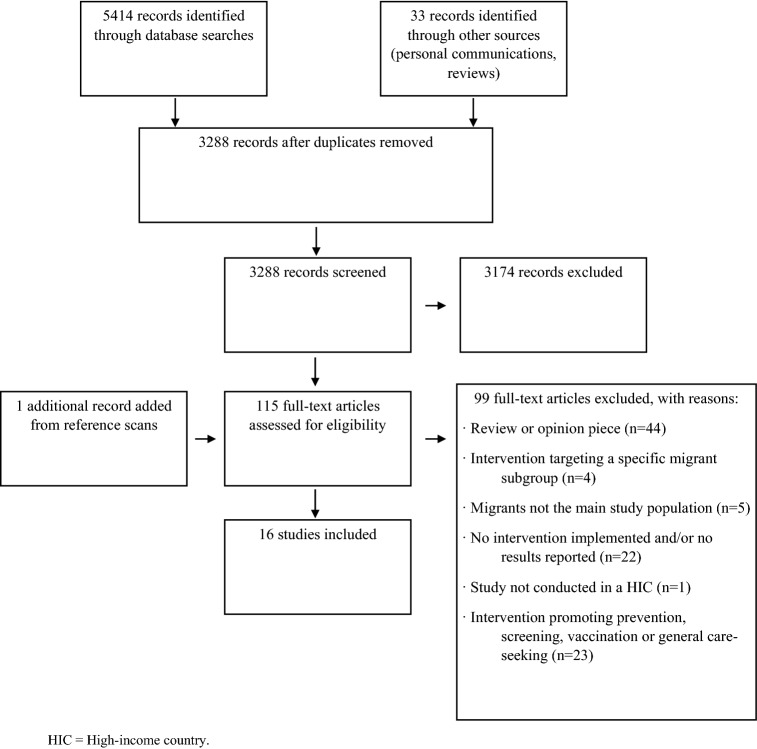
Study selection

The literature search and screening process are shown in Fig. 1. Sixteen studies were selected for inclusion, presented in Table 1.

### Characteristics of sources of evidence

All studies were conducted in North America; 15 in the USA and one in Canada. One study sought to increase care-seeking behaviour for Hepatitis B Virus (HBV) infection [28] and 15 studies for mental health problems (Table 1) [29–43]. Ten studies measured care-seeking behaviour via participants’ intent or willingness to, or their attitudes towards, care-seeking [29, 30, 35–37, 40, 43]. Six studies used care utilisation to measure care-seeking behaviour [28, 32–34, 39]. In 14 studies, outcome measures were self-reported, via surveys or interviews, while in two studies, data were extracted from clinical records (Suppl. Table 2) [33, 34].

Study population characteristics are presented in Supplementary Table 1. All studies besides three [28, 31, 43], included mainly or all female participants. One study [39] included participants aged below 17 years; the rest included participants aged 17 and over. A range of migrant groups participated in the interventions (Suppl. Table 1), and all interventions but one [39] were tailored towards an ethnic group or region of origin. The most common group was Latinxs—individuals of Latin American cultural or racial descent—or Hispanics (*n* = 7) (Suppl. Table 1) [30, 35–38, 42, 43]. Detailed information on study design and intervention characteristics is presented in Supplementary Table 2 and 3, respectively.

## Results of individual sources of evidence and synthesis

Three themes emerged during qualitative content analysis [26], corresponding to the approaches utilised to increase care-seeking: (i) health communication; (ii) support groups; and (iii) primary care-based approaches. Detailed information on the findings of the studies is presented in Table 1.

### Health communication

Health communication (the strategies used to inform, influence, and motivate audiences about health issues) [44] to increase care-seeking behaviour was tested in ten studies [28, 30, 35–38, 40–43], via three different approaches: entertainment education (EE), community outreach, and non-specific approaches. Entertainment education (EE) refers to the placement of educational content within entertainment [45]; an approach employed by five studies. Two studies [37, 43] tested the effectiveness of a fotonovela, a booklet portraying a dramatic story through pictures and captions, as a tool to increase health literacy and care-seeking for depression amongst Latinx populations. Both studies tested the fotonovela *Secret Feelings*. The intervention groups in these studies showed a marginally statistically significant, or no statistically significant, difference in the intention or willingness to seek help for depression compared to controls [37, 43].

Two studies tested the effectiveness of a psychoeducational program to increase health literacy and help-seeking for psychosis amongst Latinxs [30, 38]. Both studies reported an improvement in the likelihood that participants would recommend professional help-seeking for the symptoms of psychosis, but this was not observed in all groups (Table 1) [30, 38]. One study tested the effectiveness of a web-based transmedia intervention; over six weeks, Latinas (Latinx women) with symptoms of depression and anxiety accessed media designed to reduce reluctance to seek help for these conditions [36]. The study found that participants’ perceived confidence in their ability to, and in the importance of, seeking help were both positively associated with intention or action to seek help, post-intervention [36].

Three studies [28, 41, 42] used community outreach approaches to communicate health information and increase care-seeking. In one study, Latinas were trained as community health educators (*promotoras*) who contacted women in their community (*compañeras*) and provided them with information and coping skills for depression [42]. This study reported a statistically significant increase in positive attitudes towards depression treatment amongst *compañeras,* pre- to post-intervention [42].

Teng and colleagues implemented educational seminars about mental health for Chinese–American elders, in English and Mandarin, at a community church, and participants showed an increased inclination to seek help for psychiatric concerns following the intervention [41]. Similarly, Chao and colleagues implemented a one-day screening and informational clinic on HBV, as part of an educational outreach campaign in the Asian–American community [28]. The clinic offered free screening and physician-led educational seminars in English and Mandarin. An year later, one-third of the participants who had screened positive for chronic HBV infection reported that they had followed recommendations to seek liver cancer screening [28].

Two studies took non-specific health communication approaches to increasing care-seeking [35, 40]. One study tested the effectiveness of a brochure to increase care-seeking for suicidal thoughts amongst Latinxs, but no significant difference was observed between intervention and control groups in the change in attitude towards professional help-seeking [35]. Piwowarczyk and colleagues sought to increase care-seeking for trauma amongst East African women via a DVD-centered workshop, using African immigrant women’s stories to portray basic health information [40]. Pre- to post-intervention, there was a statistically significant increase in participants’ knowledge of, and intent to obtain, mental health care [40].

### Support groups

Weine and colleagues utilised support groups as a tool to increase care-seeking for trauma amongst Kosovar refugees [32] and PTSD amongst Bosnian-Herzegovinians [31], in two separate studies. Both interventions were based on family coping and support techniques, using multi-family group sessions to facilitate adjustment [31, 32]. Both studies reported statistically significant increases in the use of psychiatric or mental health services amongst participants who attended the support groups, compared to controls [31, 32].

### Primary care-based approaches

Four interventions took primary care-based approaches to increase migrants’ care-seeking for stigmatised conditions (Table 1) [29, 33, 34, 39]. One study piloted a self-assessment tool for psychosocial risk with Afghan refugees [29]. Three studies sought to increase migrants’ engagement with mental health services via multi-component primary care-based interventions, which sought to increase collaboration between the two services (Table 1) [33, 34, 39]. All three multi-component approaches reported some improvement in various measures of care-seeking and engagement with services following the interventions [33, 34, 39]. The self-assessment tool reported higher intention to visit a psychosocial counsellor in the intervention group compared to controls, but this was not statistically significant (Table 1) [29].

## Results in context

This scoping review mapped the evidence for interventions implemented to increase migrants’ care-seeking behaviour in high-income countries for stigmatised conditions. The relatively few included studies (*n* = 16), all from North America, indicates limited evidence within this field, while the features of reported interventions reveal several key trends. Interventions were typically tailored to one migrant group in terms of language, content, and delivery, reflecting the notion that no “one size fits all”. All interventions were community-based, indicating a trend towards local interventions. Ten interventions were administered in groups [28, 30–32, 35, 37, 38, 40, 41, 43], which can reduce costs, but fear of stigma could hinder attendance. Latinxs, Hispanics, and Asian-Americans were the most represented migrant groups in included studies (Table 1). This could indicate high burden of disease amongst these groups; it could also reflect the North American setting, or higher advocacy amongst these migrant communities than others.

Thirteen studies [28, 30–34, 36–42] reported improvements in participants’ care-seeking, while three [29, 35, 43] reported no effect (Table 1). Interventions that improved care-seeking were typically more complex and resource-intensive, which will be unpacked further in coming paragraphs. Given the stigmatisation of migrant populations [18] and the complexity of care-seeking for stigmatised conditions, these findings suggest that simple interventions may not suffice to improve this—an important finding for policy-makers and researchers.

Health communication was the most commonly used approach to increase migrants’ care-seeking [28, 30, 35–38, 40–43]; and interventions within its three subthemes (Table 1) shared common features. Information and delivery mode were tailored to the cultural preferences and literacy of target populations. A fotonovela was, for example, written at 4th-grade reading level to accommodate low literacy, and showed marginally positive effects on intent to seek care [37, 43]. Interestingly, a brochure on suicide that was not adapted to its target population’s literacy level (Table 1) [35] did not improve care-seeking. Adapting interventions to participants’ literacy level may be important to increase care-seeking, but the evidence is not conclusive.

There is a growing interest in health communication to improve health literacy and care-seeking behaviour. Low-health literacy has been documented as a barrier to migrants’ care-seeking for HIV [15] and mental health [46], thus health communication may be a promising approach to increase care-seeking for stigmatised conditions. However, designing and implementing health communication interventions (often specific to one group) can be resource intensive, requiring in-depth knowledge of target populations. In addition, the varied findings of the health communication studies reported here (Table 1) do not conclusively support the effectiveness of this type of intervention. Further research into which types of health communication are effective, for whom, and in what context, is required.

The support group interventions also sought to increase care-seeking via improving health literacy [31, 32]. Moreover, these interventions utilised a holistic approach to facilitate care-seeking, building a support network within and between families over several months. The reliability of the RCT design and extended follow-up of participants suggests that these were more resource-intensive than some of the simple health communication interventions [30, 37, 38, 41] and their conclusions more reliable. Indeed, the reported improvements to care-seeking amongst participants over a sustained period post-intervention (Table 1) suggest that further research into the efficacy of support groups in other contexts is warranted.

Primary care-based focussed on removing structural, as opposed to personal, barriers to care. The interventions within this group that successfully improved care-seeking [32, 33, 39] were multi-component; targeting several aspects of primary care (Table 1) to improve access to mental health care. The findings suggest that using primary care settings as a gateway to stigmatised condition care could be a promising avenue for further research.

This review identified several gaps in the literature. Of the 5447 hits retrieved in the literature search (Fig. 1), only 16 were selected for inclusion, all from the USA and Canada. The low number of eligible studies is surprising, so it is possible that exclusion criteria led to a selection bias. However, given the comprehensiveness of the search strategy, it is unlikely that many studies were missed. The low number of included studies likely reflects a lack of research and reporting within this field.

Given the marked increase in migration to Europe and interest in migrant health [47], the absence of European studies was surprising. Moreover, studies including minors [39] and men were underrepresented or absent, respectively. Migration is a risk factor for various mental health problems in minors [48], for example, yet research suggests that they exhibit lower care-seeking behaviour than hosting populations [49]. The lack of interventions targeting men and minors may reflect lower priority given to men, or the perceived challenges of engaging these already stigmatised groups for stigmatised conditions.

Fifteen included studies [29–43] sought to improve care-seeking for mental health problems, reflecting the rapid expansion of the migrant mental health field over recent decades [3]. Interventions that targeted other stigmatised conditions with high prevalence amongst some migrant groups, such as HIV and TB, were noticeably missing. One possible explanation is that care-seeking research for these conditions is more focussed on vaccination or treatment adherence, which were not included in this review.

Another important finding is that study design was often a limitation of included studies; half of included studies [29, 30, 36–38, 40–42] measured outcomes immediately post-intervention, which does not allow the long-term strength of effects to be measured. Six studies [30, 36, 38, 40–42] employed a one group pre/post-test design, preventing comparison with controls. Moreover, intent or attitudes towards seeking care [29, 30, 35–38, 40–43] do not necessarily translate to care utilisation, particularly for stigmatised conditions where desirability bias may be high. Fourteen studies [28–32, 35–43] used self-reported outcome measures, which further introduce desirability and recall bias. The bias introduced through study design prevents us from drawing reliable conclusions on the effectiveness of included interventions, a limitation of this review. It is also important to consider the role of the stigmatisation of migrant groups themselves, aside from the stigmatisation of the conditions, which was not explored in included studies and would likely influence findings.

This scoping review has several other limitations. Publication bias towards positive results is a common issue in intervention research. Although null findings were reported [29, 33, 35, 38, 39, 43], several of these were associated with improvements in variables besides care-seeking. Interventions that did not achieve any positive results may not been reported in the literature and thus were missed. Therefore, although not as strong as anticipated, publication bias is still a limitation of this review.

The definition of care-seeking utilised [9] does not included seeking support from informal sources, as the purpose of the review was to inform an intervention to increase care-seeking from mental health professionals. However, research has shown that these are important sources of support for many migrant communities [10], which could impact care-seeking from healthcare professionals. Further research into the relationship between these forms of care-seeking in migrant communities are needed. Similarly, improving our understanding of factors that influence low care-seeking amongst migrant populations will be critical to designing effective interventions to address this issue.

The literature search was limited to English and Swedish language articles, in the 15 included databases and key journals, thus relevant articles published in other databases and languages are likely missing. Furthermore, the review’s scope was limited by the review question and purpose. Our focus was initial care-seeking, for example, so we are unable to comment on retention of migrants in care. Further scoping reviews are needed to map the evidence in these areas. A critical appraisal of included studies was not conducted, and the heterogeneity of measured outcomes meant that it was not possible to conduct a meta-analysis, limiting comparisons between studies and interpretation of their findings.

## Conclusion

Care-seeking behaviour is a complex issue, so it is unsurprising that no “silver bullet” approach to improve care for conditions associated with stigma among migrants in high-income countries was found. Our findings do, however, reveal various trends in the field. Interventions were typically tailored to one migrant group and these were often either community- or group-based. This knowledge was used to inform the design of a multi-arm, community-based intervention, with health communication components, to improve care-seeking for mental health problems amongst migrant populations in Stockholm, Sweden. Significant gaps in the literature remain, and the included study designs were limited to discern intervention effectiveness. We recommend that future studies seek to fill these knowledge gaps, using more reliable study designs, to better inform effective policy on migrant health.

## Supplementary Information

Below is the link to the electronic supplementary material.

Supplementary file1 (DOC 47 kb)

Supplementary file2 (DOCX 84 kb)

## Data Availability

All data generated or analysed during this study are included in this published article and its supplementary information files.
