# Supplementary material for: Interventions to increase migrants’ care-seeking behaviour for stigmatised conditions: a scoping review
Source: Soc Psychiatry Psychiatr Epidemiol. 2021 Mar 29;56(6):913–30. doi: 10.1007/s00127-021-02065-1 (PMC8192321; doi:10.1007/s00127-021-02065-1)
Supplement: Supplementary file 1 — Supplementary file1 (DOC 47 kb) [file 127_2021_2065_MOESM1_ESM.doc]

**Protocol**

Arksey and O’Malley’s methodology for conducting a scoping review [1], as well as later guidance from Peters and colleagues [2], and the PRISMA-ScR extension checklist for scoping reviews [3], were used to inform this protocol. The review was conducted in five broad stages, outlined below.

Step 1 – Identify the research question

Arksey and O’Malley [1], and Peters [2], stress the importance of an iterative approach to developing a scoping review research question. VP, BN, and ACH conducted preliminary searches of the literature, and their subsequent increasing familiarity with the field helped to develop the following research question, objective and purpose:

*Research question*

Which interventions to increase migrants’ care-seeking behaviour for stigmatised conditions have been implemented in high-income countries?

Objectives

- To assess the scope of the literature available on interventions to increase migrant’s care-seeking behaviour for stigmatised conditions in high-income countries.
- To gather knowledge on intervention features and outcomes.

Purpose

The overarching purpose of the scoping review was to inform the design of an intervention to increase care-seeking behaviour from front-line mental health services amongst children and young people from migrant backgrounds in Stockholm, Sweden.

Step 2 – Identify relevant studies

*Databases*

The following electronic databases and websites, covering both peer-reviewed and grey literature, were chosen as our primary sources of information: PubMed; Web of Science; PsycINFO; Global Health; Google Scholar; Mednar; ProQuest; DART-Europe; OAIster; Bielefeld Academic Search Engine (BASE); National Center for Biotechnology Information (NCBI) Bookshelf; the World Health Organisation; and Norwegian Institute of Public Health. Furthermore, ACH and VP identified two key journals within the field of migrant health – Ethnicity & Health, and BMC Public Health – that were hand-searched to ensure that relevant studies were not missed. Finally, citation tracking (using manual searches of citations on Google Scholar) and reference scans were conducted to identify additional studies for review that could have been missed by the database and journal searches.

*Search strategy*

As recommended [1,2], VP and BN collaborated with information experts at Karolinska Institutet University Library to develop a comprehensive, iterative search strategy that was revised following new findings in the literature.

Searches of the 15 chosen electronic databases, websites, and key journals were conducted from 10/06/2019 until 05/07/2019. The search strategy for PubMed is presented in Supplementary Figure 1, which was developed as the ‘primary’ search strategy and was then adapted to other databases. The search strategies for other databases and websites are available upon request.

All search strategies were limited to studies published in English or Swedish. The search was designed to capture as much relevant literature as feasible, in line with the broad scope of the research question. However, given this broad scope and the specificity of terminology that can be used in relevant studies (for specific stigmatised conditions, for example), we recognise that relevant papers may have been missed.

Step 3 – Study selection

Search results were imported into Mendeley reference management software and screened against the eligibility criteria (defined below). VP and BN independently screened titles and abstracts of search results from all databases, websites and journals. Titles and abstracts that had no relevance to the research question, objectives, and purpose of the scoping review were discarded at this initial stage. The two reviewers met to discuss the initial screening and to agree on which studies were eligible for full-text screening. One of the reviewers, BN, had no prior experience of public health, which helped to avoid pre-formed biases.

Full-text screens of the remaining studies against eligibility criteria were conducted independently by VP and BN, who then together decided the final list of studies for inclusion, as previously described. Where VP and BN were in disagreement over whether a study met the inclusion criteria, the issue was resolved by consultation with the research group.

*Eligibility criteria*

Studies that met the following criteria were considered for inclusion: the study design included an intervention that was implemented in a high-income country; the intervention was designed to increase initial care-seeking behaviour from a healthcare professional for a stigmatised condition; and the main study population were international migrants, and/or their children.

The inclusion criteria were kept as broad as was feasible in order to effectively capture the scope of the literature within the research field. This was done via the development of a basic inclusion criteria that was updated following literature searches. For example, we had planned to include studies including any migrant population. Following the initial abstract screen, however, we noted that studies including vulnerable subgroups of migrants (such as the homeless, or men who have sex with men) reported on interventions that targeted barriers to care-seeking related to subgroup, as opposed to migrant, status. These studies were judged to be beyond the scope of the review and were excluded. Similarly, the inclusion criteria were broadened as our understanding of the literature increased. For example, studies reporting on interventions that sought to improve attitudes towards care-seeking or willingness to seek care were included, after abstract screening demonstrated that this was a commonly used measure of care-seeking. Our inclusion and exclusion criteria (including definitions informed by literature searches and discussion within the research group) are presented below.

Inclusion criteria

- **International migrant** – an individual living outside their country of birth [4]. Studies including international migrants and/or their children were eligible for inclusion in this scoping review.
- **Care-seeking behaviour** – “a problem-focused, planned behaviour, involving interpersonal interaction with a selected healthcare professional” [5]. For the purpose of this review, care-seeking behaviour was further specified as the initial act of seeking help from a healthcare professional for a stigmatised condition.
- **Changes in attitudes towards, or intention to seek, care** – changes in attitudes towards care-seeking or the intention to seek care for a stigmatised condition were commonly used measures of changes to care-seeking behaviour in the literature, thus studies measuring these outcomes were included.
- **Stigmatised conditions** – stigma is defined by the Cambridge Dictionary as “a strong feeling of disapproval that most people in society have about something”. Studies on improving care-seeking for a stigmatised condition, defined as any condition associated with stigma, were included in this scoping review.
- **High-income country** – as per the World Bank’s Classification of Countries by Income [6], from the year that each study was conducted.

Exclusion criteria

- **Low- or middle-income country** – as per the World Bank’s Classification of Countries by Income [6], from the year that each study was conducted. These studies were considered beyond the scope of the review.
- **Non-English or -Swedish language papers** - due to lack of other language abilities within the research group, these studies were excluded.
- **Care-seeking from informal sources** – defined as seeking care from non-professional sources, such as spiritual leaders and community members [7]. Although an important source of care-seeking amongst many migrant communities, informal care-seeking was deemed beyond the scope of the review and these studies were excluded.
- **Care retention** – the purpose of this review was to inform the design of an intervention to improve initial care-seeking amongst migrant children and young people, thus studies that sought to retain patients in care were beyond its scope and were excluded. For the same reason, interventions to improve adherence to a treatment regimen, or to promote screening or vaccination, were excluded.

Step 4 – Charting the data

Data was extracted from included studies and charted in a custom-built data extraction form in Microsoft Excel. This form was developed by VP and independently piloted on three included studies by VP and BN. The two reviewers then discussed the form with the wider research team, using feedback to adjust it as necessary.

The following data was extracted from included studies:

- Study characteristics: author(s), year of publication, year of data collection, location, study aim, study design, study setting (geographical and type of setting – e.g. primary care clinic).
- Study population: sampling method, sample size, age of participants, migrant status, ethnic group, sub-group specification (e.g. individuals with a specific mental health diagnosis; where applicable), gender.
- Intervention: intervention description, language of delivery, number of sessions, control group (yes/no), stigmatised condition.
- Study results: outcome measures, reported outcomes and findings related to the scoping review question (e.g. psychiatric service utilisation).

Step 5 – Collating, synthesising and reporting results

The small number of included studies and the heterogeneity of measured outcomes prevented a meta-analysis of their findings, which limited quantitative comparisons between studies and interpretation of their findings. However, data from included studies (including study characteristics, study population, and study findings) was summarised in text and table format, in order to provide a descriptive summary of the characteristics of the studies that have been conducted in this field.

Qualitative content analysis [8] was used to organise the literature according to the type of interventions implemented in order to increase care-seeking for a stigmatised condition amongst migrant populations. The included studies were sorted thematically by VP, BN, and ACH according to the approach taken within the intervention to increase care-seeking behaviour. This was an iterative process, involving discussion with the wider research group (in order to counteract and address potential biases) and continual adjustments, using the intervention type as the primary unit of analysis. Subcategories of interventions emerged within several of the developing themes thus, where applicable, interventions were further sorted into subthemes. Themes and subthemes were discussed between the researchers until a consensus was reached.

Consistent with PRISMA-ScR guidance on conducting scoping reviews [3], the methodological quality and risk of bias of the included studies were not assessed. Similarly, step six of Arksey and O’Malley’s framework, the consultation exercise [1], was not conducted as part of this scoping review but is planned for the community-based intervention that its findings have informed.

References

1 Arksey H, O’Malley L. Scoping studies: Towards a methodological framework. *Int J Soc Res Methodol Theory Pract* 2005;**8**:19–32. doi:10.1080/1364557032000119616

2 Peters MDJ, Godfrey CM, Khalil H, *et al.* Guidance for conducting systematic scoping reviews. *Int J Evid Based Healthc* 2015;**13**:141–6. doi:10.1097/XEB.0000000000000050

3 Tricco AC, Lillie E, Zarin W, *et al.* PRISMA Extension for Scoping Reviews (PRISMA-ScR): Checklist and Explanation. *Ann Intern Med* 2018;**169**:467–73. doi:10.7326/M18-0850

4 IOM, WHO, UN. International Migration, Health and Human rights. 2013. doi:10.1037/e569712006-004

5 Cornally N, Mccarthy G. Help-seeking behaviour: A concept analysis. *Int J Nurs Pract* 2011;**17**:280–8. doi:10.1111/j.1440-172X.2011.01936.x

6 Fantom N, Serajuddin U. The World Bank’s classification of countries by income. 2016. doi:10.1596/1813-9450-7528

7 Hernández-Plaza S, Alonso-Morillejo E, Pozo-Muñoz C. Social support interventions in migrant populations. *Br J Soc Work* 2006;**36**:1151–69. doi:10.1093/bjsw/bch396

8 Thomas J, Harden A. Methods for the thematic synthesis of qualitative research in systematic reviews. *BMC Med Res Methodol* 2008;**8**:1–10. doi:10.1186/1471-2288-8-45
